# Modeling and optimization of culture media for recombinant *Helicobacter pylori* vaccine antigen HpaA

**DOI:** 10.3389/fbioe.2024.1499940

**Published:** 2024-12-04

**Authors:** Runqing Tan, Song Zhou, Min Sun, Yu Liu, Xiumei Ni, Jin He, Gang Guo, Kaiyun Liu

**Affiliations:** Biopharmaceutical Research Institute, West China Hospital, Sichuan University, Chengdu, China

**Keywords:** *Helicobacter pylori*, artificial neural network, response surface methodology, recombinant antigen, rHpaA

## Abstract

**Introduction:**

*H. pylori* (*Helicobacter pylori*) infection represents a significant global health concern, exacerbated by the emergence of drug-resistant strains resulting from conventional antibiotic treatments. Consequently, the development of vaccines with both preventive and therapeutic properties has become crucial in addressing *H. pylori* infections. The *H. pylori* adhesin protein HpaA has demonstrated strong immunogenicity across various adjuvants and dosage forms, positioning it as a key candidate antigen for recombinant subunit vaccines against *H. pylori*. Optimizing fermentation culture conditions is an effective strategy to enhance product yield and lower production costs. However, to date, there has been no systematic investigation into methods for improving the fermentation yield of HpaA. Enhancing the fermentation medium to increase HpaA yield holds significant potential for application and economic benefits in the prevention and detection of *H. pylori* infection.

**Methods:**

To achieve a stable and high-yielding *H. pylori* vaccine antigen HpaA, this study constructed recombinant *Escherichia coli* expressing HpaA. The impact of fermentation medium components on the rHpaA yield was assessed using a one-factor-at-a-time approach alongside Plackett–Burman factorial experiments. Optimal conditions were effectively identified through response surface methodology (RSM) and artificial neural network (ANN) statistical computational models. The antigenicity and immunogenicity of the purified rHpaA were validated through immunization of mice, followed by Western Blot analysis and serum IgG ELISA quantification.

**Results:**

Glucose, yeast extract, yeast peptone, NH_4_Cl and CaCl_2_ all contributed to the production of rHpaA, with glucose, yeast extract, and NH_4_Cl demonstrating particularly significant effects. The artificial neural network linked genetic algorithm (ANN-GA) model exhibited superior predictive accuracy, achieving a rHpaA yield of 0.61 g/L, which represents a 93.2% increase compared to the initial medium. Animal immunization experiments confirmed that rHpaA possesses good antigenicity and immunogenicity.

**Discussion:**

This study pioneers the statistical optimization of culture media to enhance rHpaA production, thereby supporting its large-scale application in *H. pylori* vaccines. Additionally, it highlights the advantages of the ANN-GA approach in bioprocess optimization.

## 1 Introduction

*Helicobacter pylori* (*H. pylori*) is a conditional pathogen that selectively colonizes the human gastric mucosa (Wadström et al., 1996; Amieva and Peek Jr, 2016) and is closely linked to various gastrointestinal diseases (Malfertheiner, 2018; Zhou et al., 2021). *H. pylori* infection has posed a serious threat to human health for many years (Hooi et al., 2017; Li et al., 2023), with standard treatments involving triple or quadruple therapies comprising two antibiotics alongside proton pump inhibitors (PPIs) or bismuth agents (Malfertheiner et al., 2017). However, these treatments can lead to gastrointestinal microbiota dysbiosis (Karakan et al., 2021) and contribute to the development of antibiotic resistance, resulting in a continuous decline in the effectiveness of antibiotic treatment in infection eradication (Nestegard et al., 2022; Setshedi and Smith, 2023). Thus, there is an urgent need for new strategies to prevent and treat *H. pylori* infection.

Numerous studies involving the cloning of various genetic clusters or antigen genes for use in genetically engineered vaccines have been performed (Mirzaei et al., 2017; Liu et al., 2020). A pivotal 2015 study published in The Lancet reported a recombinant *H. pylori* vaccine that significantly reduced the natural infection rate in volunteers, highlighting vaccination as a promising preventive and therapeutic approach against *H. pylori* infection (Zeng et al., 2015). However, challenges remain, including the low immunogenicity and low production efficiency of antigenic components (Zhang et al., 2022; Yunle et al., 2024). Therefore, the selection and preparation of specific antigens are of great importance.

HpaA, a lipoprotein located on the surface and flagellar sheath of *H. pylori*, is a primary adhesin that binds to various receptors on gastric epithelial cells, facilitating bacterial colonization and activation and leading to gastric mucosal damage (Valkonen et al., 1997; Martini et al., 2024). HpaA is conserved across different *H. pylori* strains and is essential for colonization (Carlsohn et al., 2006). Its low homology with other *H. pylori* proteins and the strong immunogenicity make it a key candidate for vaccine development, as studies have indicated its potential to induce protective immune responses in mice (Lundström et al., 2001). While current studies have focused primarily on the immunological effects of HpaA, there is a need to optimize the production process of recombinant antigens, which is where the development and optimization of fermentation medium become crucial.

The fermentation medium defines the chemical and nutritional environment for host cells during the production of heterologous proteins and directly impacts production efficiency and economic viability (Zhang and Gtrasham, 1999). High-level expression of heterologous proteins may impose a metabolic stress on host cells, affecting their growth efficiency (Goormans et al., 2020). Adjusting the culture conditions can alleviate this stress and optimize the balance between cell growth and protein production. Considering the stringent safety standards required for vaccine components, the use of some molecular tools may increase the complexity of downstream production and purification processes, which is not conducive to industrial-scale production.

Nonetheless, a substantial body of research demonstrated that refining the nutritional parameters within the cultivation process can markedly elevate the efficiency of recombinant protein production (Su et al., 2015; Behravan and Hashemi, 2021). Among them, experimental design methods such as response surface methodology (RSM) and artificial neural network (ANN) have been successfully employed to optimize the expression and yield of recombinant proteins in host cells (Papaneophytou and Kontopidis, 2014). RSM employs polynomial functions to elucidate the interactive effects of input variables, while ANNs, structured to mimic biological neurons, identify nonlinear relationships within data (Pandey et al., 2018). Genetic algorithms (GA), grounded in evolutionary theory, have enhanced the optimization of neural networks, offering greater predictive accuracy than traditional orthogonal methods (Prabhu et al., 2017). Production of the recombinant pneumonia vaccine candidate antigen PsaA was efficiently optimized in *Escherichia coli* by RSM. The optimized medium and seed culture conditions reduced the processing time by 35% and doubled the productivity to 40 (mg/L)/h, while also decreasing the raw material costs (Larentis et al., 2012). In another study, RSM and ANN were used to predicted the specific growth rate and biomass of recombinant *Pichia pastoris* during fed-batch methanol fermentation. The feedstock control strategy for intracellular HBsAg production by *P. pastoris* was optimized, and the yield increased by 26%–31% (Beiroti et al., 2019).

To address the issues of low yield and the lack of systematic optimization of the cultivation conditions, in this study, *E. coli* BL21(DE3) was engineered to produce rHpaA, and the culture parameters were optimized via various experimental methods and statistical computational models based on RSM and ANN. The optimized production conditions increased the rHpaA yield by 93.2%, providing a reference for large-scale production. Additionally, the immunogenicity of the purified rHpaA was confirmed, and its ability to elicit a robust immune response in mice was demonstrated, underscoring its potential in the prevention and treatment of *H. pylori* infection.

## 2 Materials and methods

### 2.1 Bacterial strains and plasmids

The gene encoding the HpaA protein was derived from *H. pylori* 26,695 (GenBank accession number: AE000511.1) and optimized for *E. coli* codon usage. The *hpaA* gene, synthesized without a signal peptide by GeneCreate Biotechnology Company (Wuhan, China) was inserted into the *Nco*I and *Xho*I sites of the pET28a(+) vector, yielding the recombinant plasmid. The plasmid was transformed into *E. coli* BL21 (DE3) for protein expression. For long-term preservation, the recombinant bacterial strains were stored at −80°C in a solution containing 8% (v/v) glycerol.

### 2.2 Medium and cultivation

Luria Bertani (LB) medium, composed of 5 g/L yeast extract, 10 g/L tryptone, and 10 g/L NaCl was employed for both strain revival and liquid seed culture preparation. The glycerol stock of the recombinant *E. coli* BL21 (DE3) harboring the *rhpaA*/pET28a(+) plasmid was revived on LB agar plates at 37 °C for 16 h. A single colony was selected, inoculated into 10 mL of LB broth containing 100 μg/mL ampicillin, and incubated at 37°C and 220 rpm on a rotary shaker (Mingquan, Shanghai, China) overnight. Subsequently, 1 mL of the seed culture was diluted into 100 mL of induction media at a 1% (v/v) ratio and incubated under the same conditions until the optical density at 600 nm (OD_600_) reached 0.6–0.8. The expression of the rHpaA protein was induced by the addition of isopropyl-β-D-thiogalactoside (IPTG) to a final concentration of 0.5 mmol/L. After a 4-h induction period, the cells were harvested by centrifugation at 12,000 *g* for 5 min.

### 2.3 rHpaA yield analytical methods

The collected induced cells were suspended in phosphate-buffered saline (PBS) and sonicated. The total protein content of the lysate was quantified using the Lowry method (Lowry et al., 1951). Equal amounts of 10 μg protein were loaded for analysis by 12% sodium dodecyl sulfate‒polyacrylamide gel electrophoresis (SDS‒PAGE). After electrophoresis, the gel was stained with 0.1% Coomassie Brilliant Blue R-250 and visualized with a ChemiDoc™ MP Imaging System (Bio-Rad Laboratories, California, USA). The band areas in the SDS‒PAGE gel were quantified with ImageLab 6.1 software (Bio-Rad Laboratories, California, USA). The rHpaA yield was determined by multiplying the total protein concentration of the sample by the percentage signal intensity of rHpaA.

### 2.4 Determination of the optimum culture conditions

#### 2.4.1 One-factor-at-a-time experiment

Traditional Terrific Broth (TB) was selected as the basic culture medium for comparison. To screen effective medium components, the types of carbon source, nitrogen source, metal ions and phosphate in the medium formulation were chosen as variables, and the bacterial biomass yield and recombinant protein expression were considered as the response variables. Glucose, maltose, lactose, galactose, dextrin, sucrose, and soluble starch, each containing an equal amount of carbon content, were used to replace glycerol in the basic medium. Yeast peptone, yeast extract, beef extract, and corn flour, each with equal nitrogen content, were used as individual nitrogen sources to substitute the original mixture of yeast extract and tryptone. Furthermore, the organic nitrogen source in the TB medium was halved and replaced with an equal amount of one of the following inorganic nitrogen sources, ammonium sulfate, ammonium citrate, or ammonium chloride. To determine the effects of different metal ions on the formation of rHpaA, 1 mmol/L of Mg^2+^, Zn^2+^, Cu^2+^, Fe^2+^, Fe^3+^, Mn^2+^, Ni^2+^, Ca^2+^, or Co^2+^ was added to the basic culture medium. Additionally, the phosphate ion concentration was maintained based on the original TB composition, but the type of phosphate was changed from potassium phosphate to sodium phosphate or a mixture of both in equal concentrations. In each single screening experiment, all other factors were held constant. Statistical analysis of the quantitative results was conducted using one-way analysis of variance (ANOVA) with GraphPad Prism 9.5 software (GraphPad Software, San Diego, CA, USA). A p-value of <0.05 was considered to indicate statistical significance.

#### 2.4.2 Plackett–Burman experimental design

The Plackett–Burman design was conducted via Design Expert 13 software (Stat-Ease, Inc., Minneapolis, MN, USA) to find key factors significantly affecting rHpaA yield. The design contained six elements selected from one-factor-at-a-time experiments: glucose, NH_4_Cl, yeast peptone, yeast extract, CaCl_2_, and mixed phosphate. Each factor was set at two levels, resulting in a matrix of 12 experimental groups (see Supplementary Material) with triplicate replications to ensure the reliability of the results.

#### 2.4.3 Steepest ascent path

The steepest ascent path was utilized to guide the experimental conditions to the near-optimal region. The significant factors identified by the Plackett–Burman experiment, glucose, NH_4_Cl, and yeast extract, were optimized in steps sizes of 2, 3, and 4 based on the coefficient estimates (Table 1). Meanwhile, the remaining factors were set to their respective extreme levels, specifically with CaCl_2_ of 1 mmol/L, yeast peptone of 24 g/L, and phosphate buffer of 100 mmol/L. The highest level of rHpaA production reached was considered to be close to the center point of the experimental optimization design.

**TABLE 1:** Parameter analysis of variables in the Plackett–Burman design.

Code	Source	Coefficient estimate	Contribution (%)	F Value	p-value
	Model			17.53	0.0032
A	Glucose	−0.0164	34.3664	37.86	0.0016
B	NH_4_Cl	0.0154	30.1742	33.42	0.0022
C	CaCl_2_	−0.0073	6.8801	7.58	0.0402
D	Yeast extract	−0.0107	14.6369	16.12	0.0102
E	Yeast peptone	0.0085	9.3130	10.26	0.0239
F	Phosphates	0.0008	0.0904	0.10	0.7650

#### 2.4.4 Box–behnken design (BBD)

The BBD-RSM was employed to further optimize and determine the optimal levels of significant variables. Design Expert 13 software was utilized to conduct a three-factor, three-level experimental design. Seventeen sets of experiments, including five center points, were carried out with the highest point of the steepest ascent path as the preset center point, and the yield of rHpaA as the response value (Table 2). The second-order equation was fitted and the regression model was established as shown in Equation 1.

$$Y=\beta_{0}+\sum_{i=1}^{n}\beta_{i}x_{i}+\sum_{i=1}^{n}\sum_{j=1}^{n}\beta_{ij}x_{i}x_{j}$$ (1)

where *Y* represents the response value; *β*_*0*_ *, β*_*i*_ and *β*_*ij*_ are the equation coefficients; and *x*_*i*_ and *x*_*j*_ are the independent variables. The response optimizer of Design Expert software was used to determine the optimal values of key variables and the maximum yield of rHpaA within the experimental range.

**TABLE 2:** Steepest ascent path design.

Run	Variables			rHpaA Yield (g/L)
	Glucose (g/L)	NH_4_Cl (g/L)	Yeast extract (g/L)	
1	9	2	20	0.4775 ± 0.0312
2	7	5	16	0.5823 ± 0.0473
3	5	8	12	0.5124 ± 0.0330
4	3	11	8	0.4676 ± 0.0282
5	1	14	4	0.3012 ± 0.0307

#### 2.4.5 ANN model and GA optimization

The back propagation method was used to establish the ANN model, with the glucose, yeast extract, and NH_4_Cl concentrations in the medium as input and the rHpaA yield as the output. The transfer functions tansig and purelin were used for the hidden and output layers, respectively. The trainbr algorithm was employed to train the network. The BBD-RSM experimental data were utilized to train the neural network, with allocation ratios for the training set, test set, and validation setof 70%, 15%, and 15%, respectively. Training was halted when the mean square error (MSE) reached 1 × 10^−3^. To evaluate the fitting and predictive ability of the model, the root mean square error (RMSE), variance (R^2^) and standard error of prediction (SEP) were calculated for the established model via Equations 2–4, respectively (Mondal et al., 2023; Patruni and Rao, 2023).

$$RMSE=\sqrt{\frac{1}{n}\sum_{i=1}^{n}{\left(Y_{i,e}-Y_{i,p}\right)}^{2}}$$ (2)

$$R^{2}=1-\frac{\sum_{i=1}^{n}{\left(Y_{i,e}-Y_{i,p}\right)}^{2}}{\sum_{i=1}^{n}{\left(Y_{i,e}-{\bar{Y}}_{i,e}\right)}^{2}}$$ (3)

$$SEP=\frac{RMSE}{{\bar{Y}}_{e}}\times 100\%$$ (4)

In these formulae, *n* represents the number of samples, *Y*_*i,e*_ represents the experimental value, *Y*_*i,p*_ represents the predicted value, and “-” above a variable represents the average value of that variable.

The GA optimization approach was utilized to determine the optimal levels of significant variables in the cultivation process within the ANN model. The rHpaA yield function was employed as the fitness function for the GA, directing the iterative selection process. The program was developed using MATLAB 2023b software (MathWorks, Inc., Natick, MA, USA) along with its integrated GA toolbox.

### 2.5 Purification of rHpaA

The scale of the rHpaA/pET28a(+)/*E. coli* BL21 (DE3) cultures was increased to 1 L under the optimized culture conditions. The cells were harvested and suspended at a ratio of wet cell weight (g) to buffer volume (mL) of 1:20 in ice-cold 50 mmol/L phosphate buffer (PB, pH 7.0) supplemented with 500 mmol/L NaCl, 10 mmol/L imidazole, 100 U of benzonase and 200 mmol/L MgCl_2_. After high-pressure homogenization, the lysate was centrifuged at 12,000 *g* for 30 min. The supernatant was collected and applied to a pre-equilibrated Ni-Sepharose affinity column (Cytiva, Marlborough, MA, USA). Proteins were eluted with 50 mmol/L PB, 500 mmol/L NaCl and 100 mmol/L imidazole. The eluate was subsequently diluted 50-fold and subjected to anion-exchange chromatography using a Q-Sepharose column (Cytiva, Marlborough, MA, USA). The bound proteins were eluted with a solution of 20 mmol/L PB containing 150 mmol/L NaCl at pH 7.4 and stored at −80°C.

The purity of the purified rHpaA was assessed via size exclusion chromatography–high-performance liquid chromatography (SEC‒HPLC) on an Agilent Infinity 1260 II system (Santa Clara, CA, USA) with an Xtimate SEC-300A column (300 mm × 7.8 mm; Welch Materials Inc., Shanghai, China). The mobile phase comprised 150 mmol/L PB and 10% acetonitrile, with the flow rate of 1.0 mL/min. The column temperature was maintained at 20°C, and the injection volume was 10 µL. The protein was detected by an UV detector at 280 nm.

### 2.6 Animal immunization and sample collection

Six-week-old, specific-pathogen-free (SPF) female BALB/c mice were randomly divided into a control group and an experimental group, with 20 mice per group. The experimental group received immunization with 50 μg of rHpaA mixed with 1 mg of aluminum adjuvant (Thermo Fisher Scientific, Waltham, MA, USA) in an equal volume via intramuscular injection on days 0, 14, and 21. The control group received an equivalent treatment with PBS. Serum samples were collected from the tail veins of mice 1 week after the final immunization and isolated by centrifugation at 3,000 *g* for 5 min.

The experimental animals were purchased from Dossy Experimental Animals Co., Ltd. (Chengdu, China) and housed in the experimental animal center of West China Hospital, Sichuan University. All experiments were approved by the institutional ethics committee (number: 20220113002).

### 2.7 Assessment of antigenicity and immunogenicity

The antigenicity of rHpaA was assessed via Western blotting. The purified protein was subjected a 12% SDS-polyacrylamide gel and electrophoretically transferred onto a 0.22 μm pore-sized polyvinylidene fluoride (PVDF) membrane. The membrane was blocked with 5% (w/v) nonfat dry milk in Tris-buffered saline with 0.1% Tween-20 (TBST) for 1 h. After three washes with TBST, the membrane was incubated with either mouse anti-*H. pylori* lysate serum or anti-rHpaA serum, which was prepared in our laboratory, as the primary antibody for 2 h. Following three additional washes with TBST, the membrane was incubated with horseradish peroxidase (HRP)-conjugated goat anti-mouse IgG (Sangon Biotech, Shanghai, China) as the secondary antibody for 1 h. The SuperFemto ECL Chemiluminescence Kit (Vazyme, Nanjing, China) was used to visualize the immunoreactive bands with a ChemiDoc™ MP Imaging System.

The immunogenicity of rHpaA was evaluated using an enzyme-linked immunosorbent assay (ELISA). The wells of a 96-well ELISA plate were coated with 100 μL purified rHpaA at a concentration of 2 μg/mL and then blocked with 1% (w/v) bovine serum albumin (BSA) for 1 h at 37°C. Serial dilutions of serum samples from immunized mice were added to the wells and incubated for 2 h. Following three washes with PBST, HRP-conjugated goat anti-mouse IgG was added and incubated for 30 min at 37°C. The plate was then washed again, and 100 μL of TMB chromogenic substrate solution was added to each well for 15-min incubation at 37°C. The enzymatic reaction was stopped by adding 50 μL of 2 mol/L sulfuric acid (H_2_SO_4_) per well, and the absorbance at 450 nm was measured via a microplate reader (BioTek Instruments, Winooski, VT, USA).

## 3 Results

### 3.1 One-factor-at-a-time analysis for medium component screening

**FIGURE 1:**
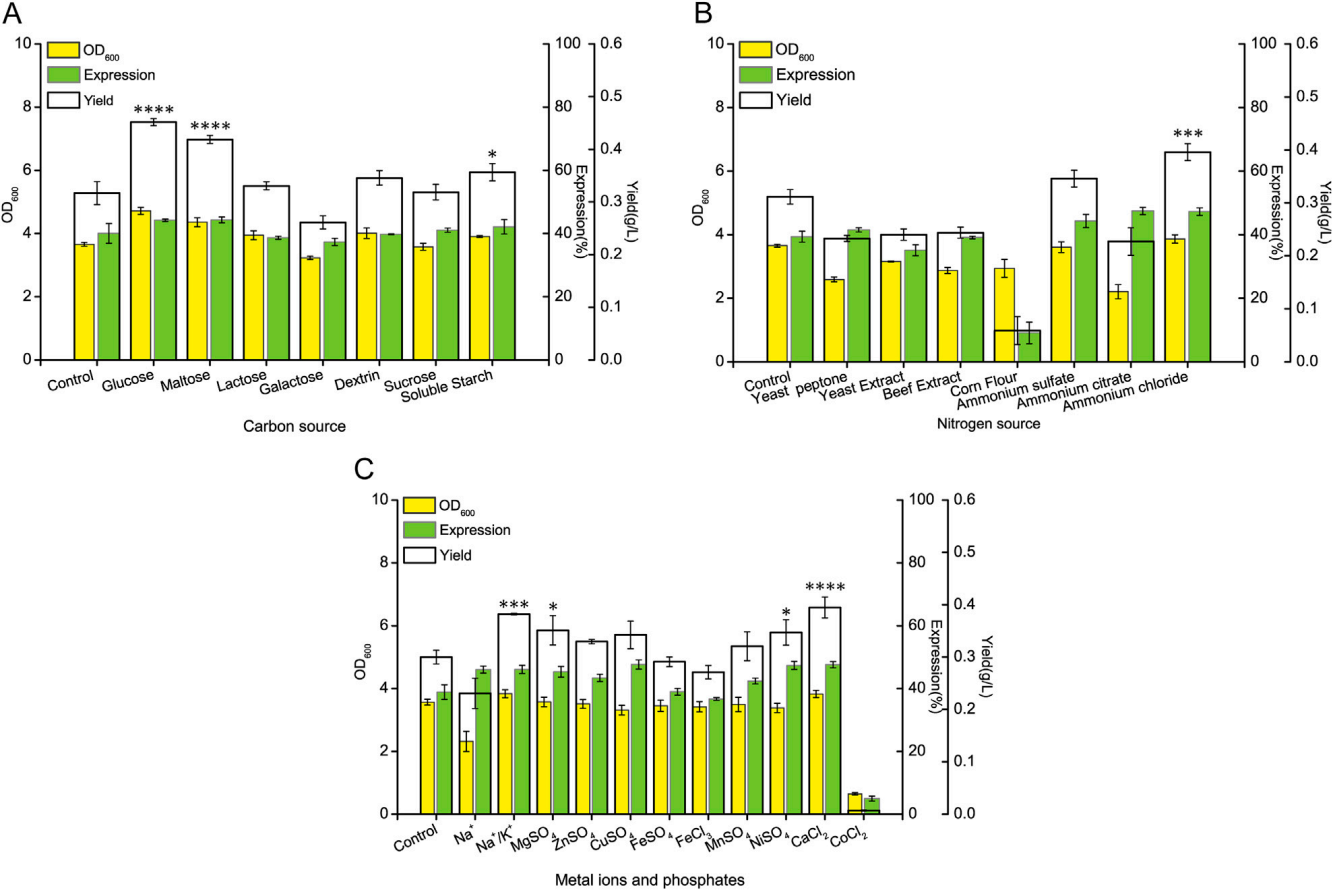
Effects of different medium components on rHpaA production. **(A)** rHpaA yield, expression rate, and OD_600_ under different carbon source conditions. **(B)** rHpaA yield, expression rate, and OD_600_ under different nitrogen source conditions. **(C)** rHpaA yield, expression rate, and OD_600_ under different phosphate and metal ion conditions. *p < 0.05, **p < 0.01, ***p < 0.001.

To enhance the yield of rHpaA, one-factor-at-a-time experiments were performed to assess the composition of the basic medium. Understanding the optimal carbon source is crucial, as it serves as the primary energy substrate for microorganisms. The experimental data (Figure 1A) showed that maltose and glucose significantly enhanced rHpaA expression, achieving levels of 44.20% and 44.33%, respectively. These levels were 10.39% and 10.71% higher than the control group. Further evaluation revealed that when glucose was used as the carbon source, resulted in the highest cell density, with an OD_600_ value of 4.72, which was 1.29-fold higher than that of the control at 3.65. The maximum rHpaA yield reached 0.45 g/L, marking a 1.41-fold increase compared to the control group.

Upon identifying the optimal carbon source, the focus shifted to the nitrogen source. The experiments (Figure 1B) revealed that the addition of inorganic nitrogen sources effectively increased rHpaA expression, but ammonium citrate inhibited cell growth. The combination of NH_4_Cl with yeast extract and yeast peptone resulted in the highest OD_600_ at 3.87 and the protein expression of 47.25%. This mix yielded 0.40 g/L of rHpaA, which was 1.27 times greater than that of the control.

Metal ions are essential for microorganisms, often serving as enzyme cofactors that influence cellular physiological processes. The next step was to investigate these elements for their potential to enhance rHpaA production. Compared to the control, the addition of Mg^2+^, Zn^2+^, Cu^2+^, Ni^2+^ and Ca^2+^ boosted rHpaA expression. Notably, only Ca^2+^ increased biomass simultaneously, resulting in an rHpaA yield of 0.39 g/L, which was 1.31 times greater than that of the control (Figure 1C).

The types of phosphate were also investigated, as they are necessary for electrochemical gradients and cellular signaling. The results indicated that the simultaneous addition of sodium phosphate and potassium phosphate to the culture medium resulted in a higher yield of rHpaA compared to the use of a single phosphate (Figure 1C). In the culture medium containing mixed phosphates, the yield of rHpaA reached 0.38 g/L, which was 1.27 times that of the control group, with an expression level of 46.06% and an OD_600_ of 3.83.

### 3.2 Plackett–Burman design for key factor identification

The Plackett–Burman experimental design is a factorial screening method used to identify key variables from a comprehensive multivariate analysis (Plackett and Burman, 1946; Quinlan and Lin, 2015). On the basis of preliminary one-factor-at-a-time experiments, six medium components were selected as variables, labeled A, B, C, D, E, and F representing glucose, NH_4_Cl, CaCl_2_, yeast extract, yeast peptone and mixed phosphate solution, respectively. The response values in the design matrix for the 12 sets of experimental conditions varied from 0.3568 to 0.4575 g/L (see Supplementary Material). Analysis with Design Expert 13 revealed that the model was statistically significant at *p* < 0.05. Among the factors, glucose (*p* = 0.0016), NH_4_Cl (*p* = 0.0022) and yeast extract (*p* = 0.0102) had the most significant effects on rHpaA yield, with contributions of 34.3664%, 30.1742% and 14.6369%, respectively (Table 1).

### 3.3 Steepest ascent path analysis for value range narrowing

The steepest ascent path is an optimization strategy for multifactorial experiments, designed to incrementally adjust factor levels towards the optimal design center. The coefficients from the Plackett–Burman experiment (Table 1) indicated that NH_4_Cl positively affecting the production of rHpaA, whereas glucose and yeast extract had negative effects. This implied that to reach the maximum response, the concentration of NH_4_Cl should be progressively increased, whereas the concentrations of glucose and yeast extract should be decreased. During the second step of the five-step ascent, the yield of rHpaA peaked at 0.585 g/L, with the concentrations of the medium components approaching the optimal solution, at which point the concentrations of glucose, NH_4_Cl, and yeast paste were 7 g/L, 5 g/L, and 16 g/L, respectively (Table 2).

#### 3.4 Model analysis for culture conditions optimization

##### 3.4.1 BBD-RSM

BBD-RSM was used to simulate the specific effects of the amounts of glucose, NH_4_Cl and yeast extract added on rHpaA production within the range of values around the peak determined by the steepest ascent path. From the design matrix of the coding variables and rHpaA production under the corresponding conditions (Table 3), the quadratic regression equation as Equation 5 was obtained.

$$\begin{array}{c} Y=-0.532804+0.084574A+0.059140B+0.093028C\\ -0.001900\text{AB}+0.001294\text{AC}-0.000673\text{BC}\\ -0.007167A^{2}-0.003260B^{2}-0.003296C^{2} \end{array}$$ (5)

where *Y* represents the yield of rHpaA (g/L), A represents glucose concentration (g/L), B represents NH_4_Cl concentration (g/L), and C represents yeast extract concentration (g/L).

**TABLE 3:** Design and results of the response surface methodology.

Run	Factors			rHpaA yield(g/L)	
	Glucose (g/L)	NH_4_Cl (g/L)	Yeast extract (g/L)	Actual response	Predicted response
1	5	2	16	0.5286 ± 0.0291	0.5234
2	9	8	16	0.5154 ± 0.0358	0.5202
3	7	5	16	0.5804 ± 0.0148	0.5914
4	9	5	20	0.4859 ± 0.0326	0.4841
5	7	5	16	0.5895 ± 0.0226	0.5914
6	7	5	16	0.5921 ± 0.0392	0.5914
7	9	5	12	0.5157 ± 0.0147	0.5175
8	5	5	12	0.5547 ± 0.0302	0.5565
9	5	8	16	0.5564 ± 0.0327	0.5613
10	9	2	16	0.5331 ± 0.0199	0.5282
11	7	5	16	0.6037 ± 0.0396	0.5914
12	7	8	20	0.4846 ± 0.0712	0.4816
13	7	8	12	0.5584 ± 0.0209	0.5518
14	5	5	20	0.4835 ± 0.0215	0.4817
15	7	5	16	0.5913 ± 0.0383	0.5914
16	7	2	20	0.4762 ± 0.0194	0.4829
17	7	2	12	0.5178 ± 0.0273	0.5209

The quadratic regression model’s ANOVA (Table 4) reveals a model p-value <0.0001, signifying statistical significance, and a lack of fit p-value >0.05, indicating an adequate fit. Thus, the model is considered both significant and sufficiently robust for the biological data analysis (Duan et al., 2020). The R^2^ value of 0.9832 suggested a strong correlation between the experimental and predicted response values. The adjusted determination coefficient (adj R^2^) value was 0.9616, indicating that 96.16% of the variation in rHpaA yield was attributed to the independent variables. From the perspective of these individual variables, yeast extract (C), glucose (A), and NH_4_Cl (B) all had significant effects on the yield of rHpaA. Additionally, the interaction between AB and AC is significant, and the contour plot is elliptical.

**TABLE 4:** ANOVA of the RSM regression model.

Source	Sum of squares	Df	Mean square	F Value	p-value
Model	0.0289	9	0.0032	45.49	< 0.0001
A-Glucose	0.0007	1	0.0007	9.43	0.0180
B-NH_4_Cl	0.0004	1	0.0004	6.22	0.0413
C-Yeast extract	0.0059	1	0.0059	82.95	<0.0001
AB	0.0005	1	0.0005	7.36	0.0301
AC	0.0004	1	0.0004	6.07	0.0433
BC	0.0003	1	0.0003	3.69	0.0961
A^2^	0.0035	1	0.0035	48.99	0.0002
B^2^	0.0036	1	0.0036	51.32	0.0002
C^2^	0.0117	1	0.0117	165.83	<0.0001
Residual	0.0005	7	0.0001		
Lack of fit	0.0002	3	0.0001	1.03	0.4689
Pure error	0.0003	4	0.0001		
Cor total	0.0294	16			

**FIGURE 2:**
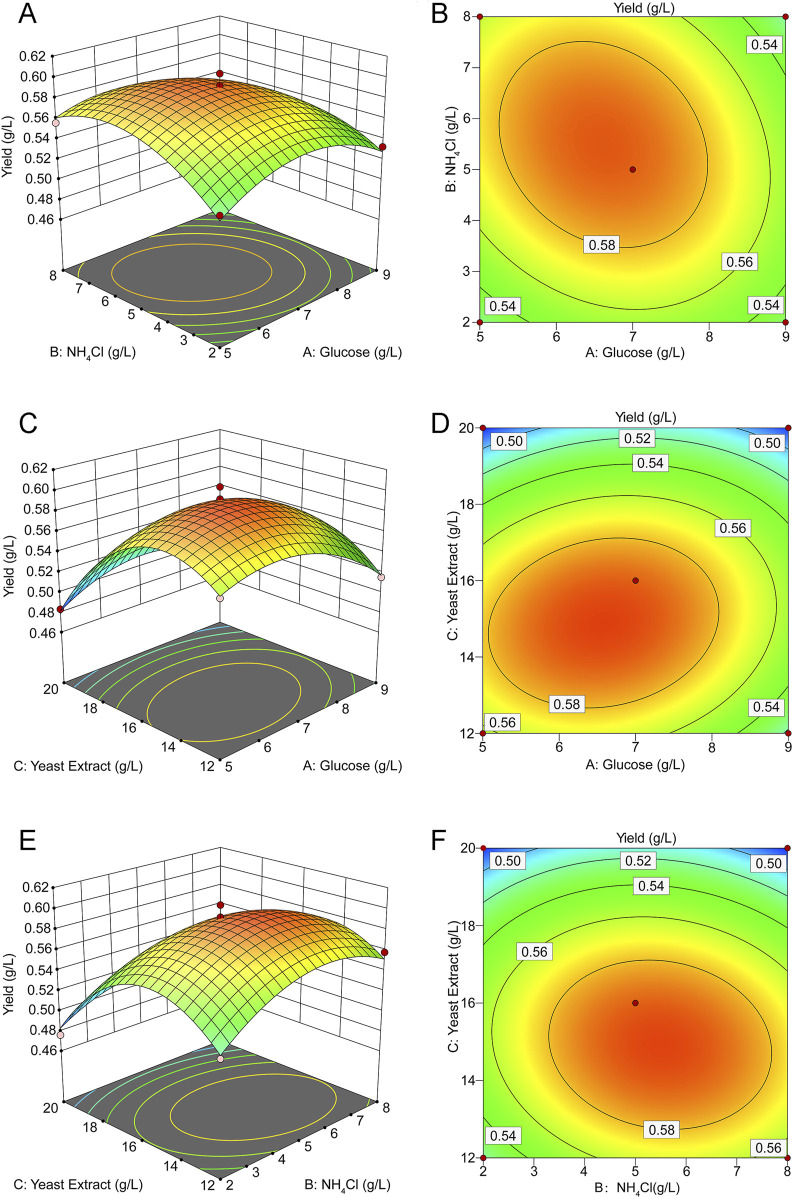
Response surface and contour plot for rHpaA production. **(A)** Response surface of NH_4_Cl and glucose concentrations on rHpaA yield. **(B)** Contour plot of NH_4_Cl and glucose concentrations on rHpaA yield. **(C)** Response surface of yeast extract and glucose concentrations on rHpaA yield. **(D)** Contour plot of yeast extract and glucose concentrations on rHpaA yield. **(E)** Response surface of yeast extract and NH_4_Cl concentrations on rHpaA yield. **(F)** Contour plot of yeast extract and NH_4_Cl concentrations on rHpaA yield.

The three-dimensional response surface and contour plots (Figure 2) visually demonstrate the effects of the different variables on the response values in the regression equation. The three sets of variables showed the same response trend: when one variable remained unchanged, the response value initially increased and then decreased as the other two variables increased. This indicated that the yield of rHpaA has a maximum value on the surface. The response optimizer of Design Expert software was utilized to maximize rHpaA yield within a defined range. At glucose, NH_4_Cl, and yeast extract concentrations of 6.488 g/L, 5.653 g/L, and 14.807 g/L, respectively, the RSM forecasted a peak rHpaA yield of 0.597 g/L. Three parallel experiments were carried out under the optimal conditions to verify the optimized results. The yield of rHpaA was 0.5878 ± 0.0157 g/L (n = 3), which was 88.4% higher than the basic medium.

##### 3.4.2 ANN-GA

**FIGURE 3:**
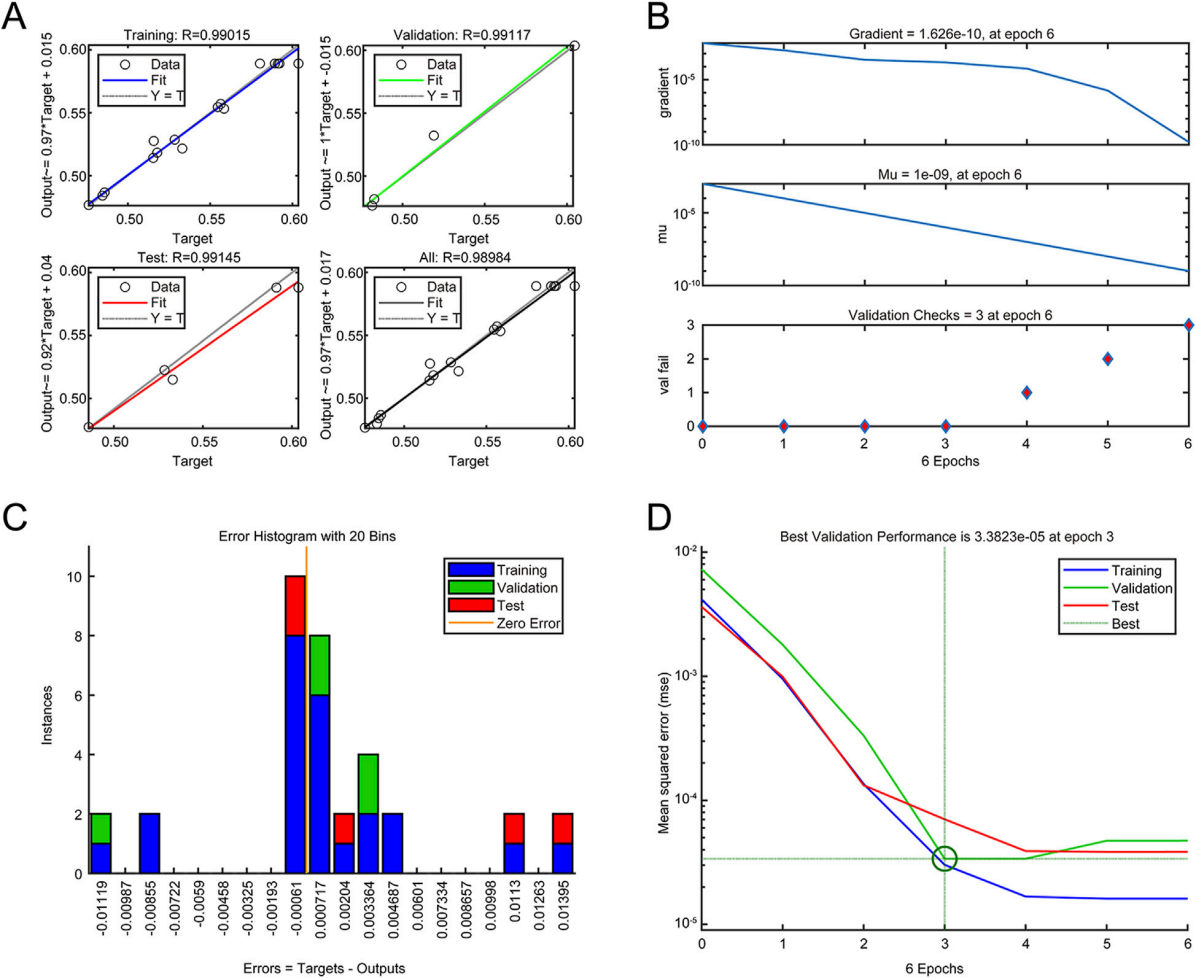
ANN model analysis. **(A)** Data regression plot. **(B)** Training state plot. **(C)** Error histogram. **(D)** Training performance plot.

To develop more precise and advanced optimization strategies, the following sections were dedicated to the application of the ANN and GA. The neural network was trained with the data from the BBD, and the number of neurons in the middle layer was determined to be 6 via trial and error process; thus, the optimal topology of the neural network was determined to be 3-6-1. The training performance plot (Figure 3D) and the training state plot (Figure 3B) showed a progressive reduction in model error with each iteration. At the third iteration, the MSE reached its nadir, signifying model stabilization and minimal predictive error. The error histogram (Figure 3C) closely adhered to a normal distribution centered near zero, indicating the absence of systemic prediction biases and high predictive accuracy. The fitting plots for the training, testing, and validation data (Figure 3A) exhibited a high degree of data fitting, with an R^2^ value of 0.9895, suggesting the robust generalizability of the model. Therefore, the ANN model is capable of accurately predicting the rHpaA yield, demonstrating high precision and low error rates.

**FIGURE 4:**
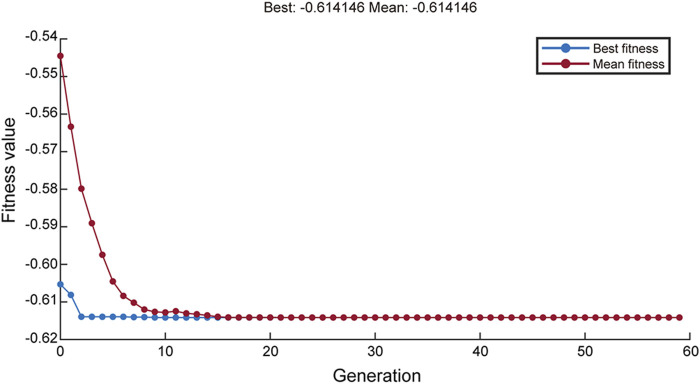
ANN-GA fitness function evolution of rHpaA production.

GA was applied to the aforementioned neural network model for global training, with iterative repetitions to achieve the maximum yield of rHpaA. After 58 rounds of GA optimization, the average fitness value and optimal fitness value of the objective function approached the same value (Figure 4), yielding a theoretical maximum yield of 0.614 g/L, with the optimal medium composition comprising 6.492 g/L glucose, 6.655 g/L NH_4_Cl, and 14.910 g/L yeast extract. Under these cultivation conditions, the rHpaA yield obtained from three replicate experiments was 0.6123 ± 0.0391 g/L (n = 3), which was 93.2% higher than that of the initial basal medium.

##### 3.4.3 Comparison of the RSM and ANN models

**FIGURE 5:**
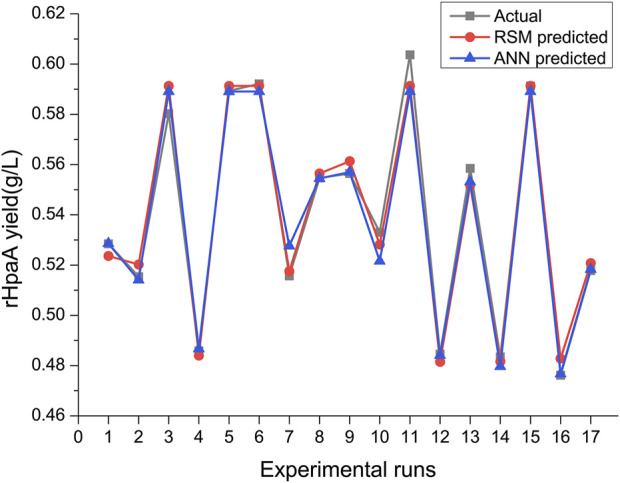
Comparison of the experimental values and the predicted values from the BBD and ANN.

Optimization and prediction through two models have led to the determination of the optimal medium formulation for rHpaA production. To facilitate a direct comparison, parameters for the RSM and ANN models were calculated. Both models exhibited strong fitting capabilities, with R^2^ values exceeding 0.95. The ANN model demonstrated superior predictive accuracy and data fitting, with RMSE and SEP values of 5.496 × 10^−3^ and 1.021%, respectively, which are lower than those of the RSM model at 5.66 × 10^−2^ and 10.49%. More intuitively, the scatter plot of data comparison (Figure 5) clearly showed the predicted results of the ANN model are closer to the experimental values. Under the two optimal culture conditions, the predicted yield from the RSM model differed from the experimental yield by 1.57%, while the predicted yield from the ANN-GA model differed from the actual yield by 0.28%, showing a smaller deviation of the ANN-GA predictions from the experimental values.

#### 3.5 Purification and identification of rHpaA

**FIGURE 6:**
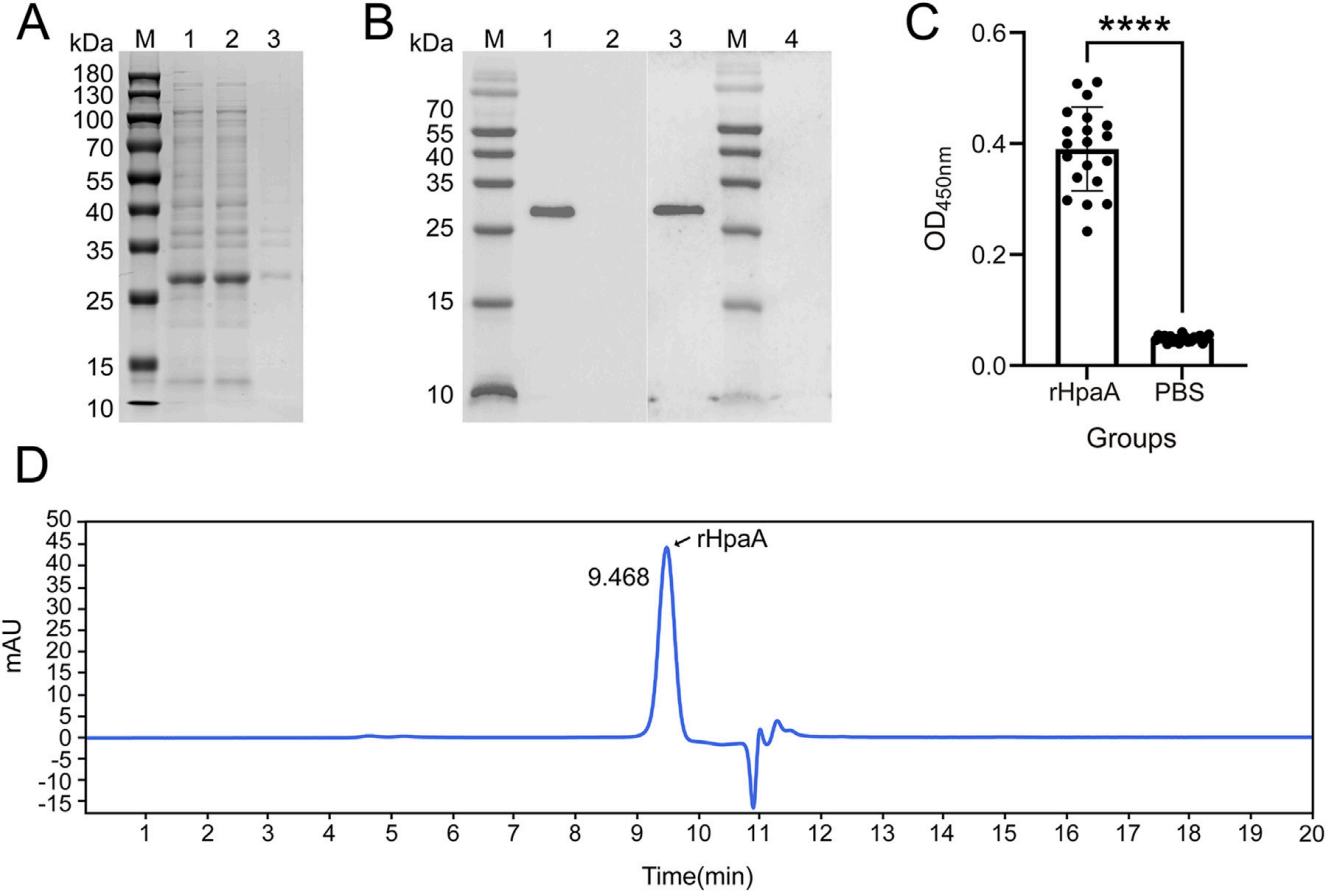
Identification and analysis of purified rHpaA. **(A)** Expression analysis of rHpaA via SDS‒PAGE. Lane M, molecular weight marker. Lane 1; rHpaA in whole bacteria; Lane 2, rHpaA in the supernatant; Lane 3, rHpaA in the precipitate. **(B)** Western blot analysis of purified rHpaA. Lane M, molecular weight marker; Lane 1, reactivity bands of purified rHpaA with anti-rHpaA serum; Lane 2, blank control for Lane 1; Lane 3, reactivity bands of purified rHpaA with anti-H. pylori lysate; Lane 4, blank control for Lane 3. **(C)** Serum-specific IgG analysis of the rHpaA-immunized group and the PBS control group. **(D)** SEC-HPLC analysis of purified rHpaA.

The optimal cultivation conditions determined with the ANN-GA model were upscaled, and the cell pellets were harvested and lysed. The rHpaA was expressed in a soluble form in the optimized medium (Figure 6A). Upon lysis, the supernatant was separated, and the target protein was subjected to sequential purification steps involving Ni^2+^ affinity chromatography and anion-exchange chromatography. The purity and homogeneity of the final rHpaA preparation were assessed by SEC-HPLC, which revealed a distinct peak at a retention time of 9.468 min (Figure 6D), indicating a high degree of purity and uniformity.

To validate the application value of rHpaA, an immunization study was conducted in mice via intramuscular injection. Western blotting was performed to evaluate the antigenicity of rHpaA, using mouse anti-*H. pylori* lysate serum or anti-rHpaA serum as the primary antibody. There was a single band at approximately 28 kDa (Figure 6B), revealing both samples specifically reacted with rHpaA. The recombinant antigen can be recognized by the mouse anti-*H. pylori* lysate serum.

ELISA was conducted to evaluate the immunogenicity of rHpaA. After serial dilution up to 262,144-fold, the levels of specific IgG in the anti-rHpaA serum remained significantly higher than those in the negative control (PBS group), with a statistically significant difference (*p* < 0.0001) (Figure 6C).

### 4 Discussion

The current study systematically investigated the effects of various culture medium components on rHpaA production by recombinant *E. coli* for the first time, and the optimal component concentration of the medium was determined by RSM and ANN model optimization. It also explored the purification methods and the fundamental immunological properties of rHpaA.

Different culture medium compositions may exert varying effects on microbial growth and product metabolism. The results of one-factor-at-a-time experiments indicated that utilizing glucose as the carbon source significantly enhanced the expression efficiency of the recombinant *E. coli*, leading to a marked increase in rHpaA production. The preference for glucose may be attributed to its rapid uptake and metabolism by bacteria, which facilitated more efficient energy conversion (Jeckelmann and Erni, 2020). Furthermore, this study demonstrated that the combined use of inorganic and organic nitrogen sources is more advantageous for rHpaA production compared to the use of a single nitrogen source. Notably, the addition of NH_4_Cl resulted in a significant increase in rHpaA production. Inorganic nitrogen sources provide more quick-acting nitrogen and reduce the lag phase, thereby promoting rapid strain growth and metabolism (Kuypers et al., 2018). Replacing complex organic nitrogen sources like yeast extract and peptone with inorganic nitrogen sources also helps control production costs for economic feasibility (Terol et al., 2019). The addition of Ca^2+^ and the use of a mixed phosphate significantly enhanced the production of rHpaA. Ca^2+^ can affect ribosome function and protein folding (Ohkuri et al., 2002). The phosphate mixture can supply both K^+^ and Na^+^, providing greater stability in maintaining ion balance and osmotic pressure across the cell membrane, thereby better supporting the transport of nutrients into cells (Stautz et al., 2021; Szatmári et al., 2020).

Microbial metabolite synthesis critically depends on the nutritional environment, with different culture media causing significant changes in protein expression, primarily due to variations in resource allocation (Goormans et al., 2020). Considering the interaction and complex effects of multiple factors in the engineered *E. coli* culture medium, multi-factor optimization methods were used to avoid deviation from the results of one-dimensional studies. Plackett–Burman experimental analysis identified glucose, yeast extract, and NH_4_Cl as the most significant variables, which are essential carbon and nitrogen sources that greatly influence rHpaA as direct energy substrates. The steepest ascent design further determined the value range of the central composite design, promoting the transition from preliminary variable screening to a more detailed optimization process, and prepared for subsequent precise adjustments (Sztendur and Diamond, 2002). The RSM model established a quadratic regression equation relating actual variables to rHpaA yield, with a three-dimensional plot highlighting significant interactions between glucose and yeast extract, as well as glucose and NH_4_Cl (Jankovic et al., 2021). Utilizing RSM data, the ANN model further dissected the implicit nonlinear relationships of input variables on rHpaA yield. With GA optimization, a definitive formula for the culture medium that significantly enhances rHpaA production was derived. Concurrently, the ANN-GA model exhibited superior regression accuracy in both the prediction and optimization process (Prabhu et al., 2017). The limitation of BBD-RSM is its capacity to construct only polynomial regression models, which restricts its generalization ability when addressing complex data sets (Hamza et al., 2024). In contrast, artificial neural networks possess a nonlinear activation function that effectively captures intricate nonlinear relationships within the data, thereby providing enhanced data processing and generalization capabilities (Liu et al., 2022). This makes neural networks more suitable for guiding the optimization process of biologics manufacturing.

The structure and immunogenicity of rHpaA were critical factors influencing the protective efficacy of the vaccine. SEC-HPLC results indicated that rHpaA, following two-step purification, exhibited high purity and homogeneity. The recombinant antigen was recognized by mouse anti-*H.pylori* lysate serum, demonstrating that the rHpaA possessed antigenicity comparable to that of the natural HpaA from *H. pylori* (Lundström et al., 2003). The quantitative analyses of specific IgG in the serum of immunized mice revealed that the rHpaA antigen displayed high immunogenicity and effectively induced specific IgG responses, thereby confirming its potential for vaccine application (Banga Ndzouboukou et al., 2021).

In the development of *H. pylori* vaccines, HpaA is widely regarded as one of the most promising antigens. While most previous studies have concentrated on the immune effects and mechanisms of HpaA, there has been less focus on its engineering applications. To achieve large-scale application in the vaccine field, it is crucial to systematically enhance the antigen production yield. The optimization of fermentation conditions is a key strategy to significantly improve both product yield and quality. This study successfully increased rHpaA production through a series of experiments and model design methodologies, establishing a foundation for the future commercialization of *H. pylori* vaccines composed of rHpaA. Despite these positive findings, it is important to acknowledge the limitations of this study, which primarily focused on optimizing the composition of the culture medium. Future research must tackle the challenge of refining a broader range of culture conditions during scale-up fermentation to ensure improved scalability and reproducibility of the production method.

### 5 Conclusion

This study applied a systematic optimization strategy for the culture medium, in conjunction with RSM and ANN, leading to a significant enhancement in the production of rHpaA. The obtained rHpaA exhibited robust immunogenicity in animal models, providing a viable solution for its large-scale production in *H. pylori* vaccines. The ANN-GA model demonstrated superior accuracy in prediction and optimization, underscoring its broad utility in biotechnology.

## Data Availability

The original contributions presented in the study are included in the article/Supplementary Material, further inquiries can be directed to the corresponding authors.
