# Supplementary material for: Modeling and optimization of culture media for recombinant Helicobacter pylori vaccine antigen HpaA
Source: Front Bioeng Biotechnol. 2024 Dec 4;12:1499940. doi: 10.3389/fbioe.2024.1499940 (PMC11652157; doi:10.3389/fbioe.2024.1499940)
Supplement: Supplementary file 1 [file Table1.docx]

**Supplementary Materials**

**Table S1.** Experimental factors and levels of Plackett–Burman design.

| Code | Factor | Low Level (−1) | High Level (+1) |
| --- | --- | --- | --- |
| A | Glucose(g/L) | 5 | 20 |
| B | NH_4_Cl (g/L) | 2 | 8 |
| C | CaCl_2_ (mmol/L) | 1 | 4 |
| D | Yeast extract (g/L) | 12 | 48 |
| E | Yeast peptone (g/L) | 6 | 24 |
| F | Phosphates (mmol/L) | 25 | 100 |

**Table S2.** Encoded variable design matrix of Plackett–Burman experiment.

| Run | A | B | C | D | E | F | rHpaA yield(g/L) |
| --- | --- | --- | --- | --- | --- | --- | --- |
| 1 | 1 | -1 | 1 | 1 | -1 | 1 | 0.3568±0.0219 |
| 2 | -1 | 1 | -1 | 1 | 1 | -1 | 0.4510±0.0207 |
| 3 | -1 | 1 | 1 | -1 | 1 | 1 | 0.4575±0.0457 |
| 4 | 1 | 1 | 1 | -1 | -1 | -1 | 0.4001±0.0210 |
| 5 | -1 | -1 | -1 | -1 | -1 | -1 | 0.4155±0.0170 |
| 6 | -1 | -1 | -1 | 1 | -1 | 1 | 0.4047±0.0251 |
| 7 | 1 | -1 | 1 | 1 | 1 | -1 | 0.3712±0.0199 |
| 8 | 1 | 1 | -1 | 1 | 1 | 1 | 0.4128±0.0220 |
| 9 | -1 | 1 | 1 | 1 | -1 | -1 | 0.4287±0.0113 |
| 10 | -1 | -1 | 1 | -1 | 1 | 1 | 0.4307±0.0271 |
| 11 | 1 | -1 | -1 | -1 | 1 | -1 | 0.4178±0.0459 |
| 12 | 1 | 1 | -1 | -1 | -1 | 1 | 0.4317±0.0288 |

**Table S3.** Experimental factors and levels of the BBD.

| Factors | Code | Experimental Levels | | |
| --- | --- | --- | --- | --- |
|  |  | −1 | 0 | +1 |
| Glucose (g/L) | A | 5 | 7 | 9 |
| NH_4_Cl (g/L) | B | 2 | 5 | 8 |
| Yeast extract (g/L) | C | 12 | 16 | 20 |
